# Chronic Inflammatory Demyelinating Polyneuropathy Presenting With Isolated Distal Fingertip Paresthesia in a Factory Worker With Ulcerative Colitis: A Case Report

**DOI:** 10.7759/cureus.97218

**Published:** 2025-11-19

**Authors:** Albert Gassull

**Affiliations:** 1 Emergency Medicine, Hospital Universitari Sant Joan de Reus, Tarragona, ESP

**Keywords:** autoimmune, carpal tunnel exclusion, case report, diabetes, distal fingertips, occupational health, paresthesia, peripheral neuropathy, sensory loss, ulcerative colitis

## Abstract

Peripheral neuropathy (PN) is an underrecognized extraintestinal manifestation of inflammatory bowel disease (IBD), including ulcerative colitis (UC). We report a case of a 52-year-old male patient with UC and type 2 diabetes mellitus who developed progressive weakness and sensory disturbances localized to the fingertips of both hands over approximately one year. Initial evaluation by occupational health services did not identify any occupational cause, and subsequent public health assessments yielded no further explanation. Upon presentation to our clinic, examination revealed diminished sensation in the distal phalanges and mild grip weakness, without lower limb involvement. Laboratory testing excluded common metabolic and nutritional causes. Electromyography and nerve conduction studies confirmed demyelinating polyneuropathy consistent with chronic inflammatory demyelinating polyneuropathy (CIDP). The patient was treated with intravenous immunoglobulin, which led to a marked improvement in symptoms, with sustained remission during follow-up. This case demonstrates an atypical sensory-predominant CIDP variant restricted to the upper extremities in the context of UC. It highlights the importance of considering autoimmune neuropathies in IBD patients presenting with unusual neuropathic symptoms and underscores the need for comprehensive neurologic evaluation to prevent diagnostic delays and optimize patient outcomes.

## Introduction

Inflammatory bowel diseases (IBD), such as ulcerative colitis (UC), are chronic autoimmune conditions primarily affecting the gastrointestinal tract but often manifesting extraintestinally in joints, skin, eyes, and the nervous system. Neurological complications occur in up to 3%-5% of IBD cases, with peripheral neuropathy (PN) being one of the less common yet impactful manifestations [1]. PN in IBD can present as axonal or demyelinating forms, sometimes linked to nutritional deficiencies, medication toxicities (e.g., from metronidazole or anti-TNF (tumor necrosis factor) agents), or direct autoimmune attacks on peripheral nerves [2,3]. Among them, chronic inflammatory demyelinating polyneuropathy (CIDP) is a rare but treatable autoimmune neuropathy characterized by progressive weakness and sensory loss over at least eight weeks, often responding to immunomodulatory therapies [4].

The association between UC and PN is underreported, with prevalence estimates varying from 0.7% to 3.6% in cohort studies, potentially due to overlapping symptoms with other comorbidities like diabetes or occupational exposures [5,6]. In patients with UC, PN may arise from immune-mediated mechanisms, including molecular mimicry or cross-reactivity involving gut microbiota and neural antigens, leading to demyelination or axonal damage [7]. Comorbid diabetes adds diagnostic complexity, as diabetic neuropathy typically involves lower limbs first, whereas IBD-related PN can exhibit atypical patterns, such as isolated upper limb involvement [8].

Occupational factors, particularly in manual laborers like factory workers, may exacerbate or mimic neuropathic symptoms, often leading to initial misattribution to conditions like carpal tunnel syndrome (CTS). In Spain, workers' compensation systems (Mutuas) frequently evaluate such complaints, but non-occupational etiologies can result in diagnostic oversights [9]. This case is noteworthy for its unusual presentation of sensory symptoms confined to fingertip paresthesia without lower extremity involvement, despite concurrent diabetes, highlighting a potential predominant role of UC-driven autoimmunity. Reporting such cases is crucial to raise awareness, as early diagnosis and treatment can prevent irreversible nerve damage and improve the patient's quality of life. We present a patient whose delayed diagnosis underscores systemic healthcare gaps in addressing multifactorial neuropathies in IBD.

## Case presentation

A 52-year-old male factory worker with a 10-year history of UC and type 2 diabetes mellitus presented to our occupational medicine clinic in October 2024 with a one-year history of progressive bilateral hand weakness and sensory loss. The patient, employed in assembly line work involving repetitive manual tasks, first noticed the symptoms in September 2023, including numbness and tingling limited to the tips of his fingers, accompanied by reduced grip strength that interfered with tool handling. He denied trauma, excessive alcohol consumption (less than two units per week), smoking, or recent infections. His UC was managed with mesalazine 2.4 g/day, in remission for three years without flares, and diabetes was controlled with metformin 1000 mg twice daily, without prior neuropathic symptoms in his feet or elsewhere.

Clinical findings upon presentation

The patient reported no radiation to forearms, no neck pain, and intact lower limb sensation and strength. Physical examination revealed symmetric diminished pinprick and vibration sense in the distal phalanges of digits 1-5 bilaterally, with preserved two-point discrimination proximally. Grip strength was mildly reduced (4/5 bilaterally), but reflexes were normal (2+), and no muscle atrophy or fasciculations were noted. Foot exam was unremarkable, with normal pedal pulses and sensation. No signs of active UC, such as abdominal pain or diarrhea, were present.

Timeline

*September 2023:* Onset of bilateral fingertip numbness, tingling, and reduced grip strength.

*November 2023:* Evaluation by Mutua (workers' compensation); electrophysiological studies ruled out carpal tunnel syndrome, deemed non-work-related; referred to public healthcare, where symptoms were attributed to possible diabetic neuropathy with symptomatic analgesics prescribed.

*October 2024:* Presentation to our clinic due to worsening symptoms affecting fine motor tasks; comprehensive evaluation initiated, including laboratory tests and repeated electrophysiology.

Diagnostic assessment

Initial occupational assessment in November 2023 included electrophysiological studies showing normal median nerve latencies across the wrist but mild slowing in distal segments, ruling out carpal tunnel syndrome. Upon re-evaluation in our clinic, comprehensive blood tests were performed to investigate an autoimmune etiology given his UC history. The key laboratory findings are summarized in Table 1.

**Table 1:** Selected Laboratory Investigations at Presentation

Test	Result	Reference Range	Interpretation
Hematology			
Hemoglobin	14.2 g/dL	13.5-17.5 g/dL (males)	Normal
White Blood Cell Count	7.8×10³/μL	4.0-11.0×10³/μL	Normal
Platelets	256×10³/μL	150-450×10³/μL	Normal
Inflammatory Markers			
Erythrocyte Sedimentation Rate (ESR)	52 mm/h	<20 mm/h	Elevated (suggests inflammation)
C-Reactive Protein (CRP)	18 mg/L	<5 mg/L	Elevated (mild systemic inflammation)
Diabetes Control			
Glycated Hemoglobin (HbA1c)	6.8%	<5.7% (normal); 5.7-6.4% (prediabetes)	Controlled diabetes, unlikely acute diabetic neuropathy driver
Fasting Glucose	112 mg/dL	70-99 mg/dL	Mildly elevated, consistent with known type 2 diabetes (T2DM)
Nutritional/Toxic			
Vitamin B12	456 pg/mL	200-900 pg/mL	Normal (rules out deficiency neuropathy)
Folate	12 ng/mL	>5.4 ng/mL	Normal
Autoimmune/Endocrine			
Thyroid-Stimulating Hormone (TSH)	2.1 mIU/L	0.4-4.0 mIU/L	Normal (rules out hypothyroidism)
Rheumatoid Factor (RF)	Negative	Negative	Rules out rheumatoid arthritis (RA)-associated neuropathy
Antinuclear Antibody (ANA)	Negative	Negative	Rules out systemic autoimmune overlap
Anti-Cyclic Citrullinated Peptide (anti-CCP)	Negative	Negative	Rules out RA
Neuropathy-Specific Serology			
IgM Anti-GM1 Antibodies	1:1600 (positive)	<1:200 (negative)	Elevated; supports autoimmune demyelinating neuropathy (e.g., chronic inflammatory demyelinating polyneuropathy (CIDP) variant)
Other (Toxicology Screen)			
Heavy Metals (Lead, Mercury)	Negative	Negative	Rules out occupational toxic neuropathy

The results included hemoglobin at 14.2 g/dL, white blood cell count of 7.8x10^9^/L, platelets at 256x10^9^/L, erythrocyte sedimentation rate (ESR) at 52 mm/h (elevated), C-reactive protein (CRP) at 18 mg/L (elevated), glycated hemoglobin (HbA1c) at 6.8%, fasting glucose at 112 mg/dL, vitamin B12 at 456 pg/mL (normal), folate at 12 ng/mL (normal), thyroid-stimulating hormone (TSH) at 2.1 mIU/L (normal), and negative rheumatoid factor, anti-nuclear antibody (ANA), and anti-CCP (cyclic citrullinated peptide). Notably, serological assay for anti-ganglioside antibodies was positive for immunoglobulin M (IgM) anti-GM1 at 1:1600.

Electromyography and nerve conduction studies were repeated, revealing demyelinating features: prolonged distal motor latencies and temporal dispersion without axonal loss. Sensory nerve action potentials were reduced in the distal upper limbs but preserved elsewhere. These electrodiagnostic findings, characteristic of CIDP, are illustrated in Tables 2, 3, showing representative examples of the demyelinating patterns observed. Lumbar puncture was declined by the patient, but the clinical presentation and electrodiagnostic results met the definite diagnostic criteria of the 2021 European Academy of Neurology/Peripheral Nerve Society (EAN/PNS) guidelines for CIDP, which allow for diagnosis without cerebrospinal fluid analysis when electrophysiology is confirmatory. Diagnostic challenges included differentiating from diabetic neuropathy, given the comorbidity, but the atypical upper-limb-restricted distribution, demyelinating pattern (versus typical axonal in diabetes), and absence of lower limb involvement favored CIDP. Other diagnoses considered included entrapment neuropathies (ruled out) and nutritional/toxic causes (negative labs). Prognostic characteristics suggested a favorable response to immunomodulation based on the demyelinating nature.

**Table 2:** Motor Nerve Conduction Study Results ms: Milliseconds (latency) Δt (ms): Time difference between wrist and elbow latency m/s: Meters per second (conduction velocity); cm: Centimeters (distance) O-P Amplitude (mV): Onset-to-peak amplitude in millivolts L: Left; R: Right.

Nerve	Side	Onset Wrist (ms)	Onset Elbow (ms)	Δt (ms)	Conduction Velocity (m/s)	Distance (cm)	O-P Amplitude (mV)	Reference Normal Values
Median	L	16.3	24.0	7.7	38.0	29.3	3.8	Wrist onset ≤12 ms; Velocity 50-60 m/s; Amplitude 4-10 mV
Median	R	16.8	24.2	7.4	39.2	28.9	3.5	Wrist onset ≤12 ms; Velocity 50-60 m/s; Amplitude 4-10 mV
Ulnar	L	17.5	23.2	5.7	42.0	23.9	3.0	Wrist onset ≤14 ms; Velocity 50-65 m/s; Amplitude 3-8 mV
Ulnar	R	16.2	21.4	5.2	42.7	22.3	2.8	Wrist onset ≤14 ms; Velocity 50-65 m/s; Amplitude 3-8 mV

**Table 3:** Sensory Nerve Conduction Study Results ms: Milliseconds (latency) m/s: Meters per second (conduction velocity) cm: Centimeters (distance) SNAP Amplitude (µV): Sensory nerve action potential amplitude in microvolts L: Left; R: Right

Nerve	Side	Peak Latency (ms)	Conduction Velocity (m/s)	Distance (cm)	SNAP Amplitude (µV)	Reference Normal Values
Median	L	3.9	38.0	15.3	12.1	Peak Latency≤3.5 ms; Velocity 50-60 m/s; Amplitude 15-50 µV
Median	R	4.1	39.2	15.0	11.3	Peak Latency≤3.5 ms; Velocity 50-60 m/s; Amplitude 15-50 µV
Ulnar	L	4.2	42.0	14.0	10.1	Peak Latency≤3.8 ms; Velocity 50-65 m/s; Amplitude 10-40 µV
Ulnar	R	4.1	42.7	13.5	9.3	Peak Latency ≤ 3.8 ms; Velocity 50-65 m/s; Amplitude 10-40 µV

Therapeutic intervention

Treatment was initiated with intravenous immunoglobulin (IVIG) at 0.4 g/kg/day for five days, followed by prednisone 1 mg/kg tapered over eight weeks. No changes in therapeutic interventions were required beyond transitioning to maintenance IVIG.

Follow-up and outcomes

At four-week follow-up, paresthesia improved by 70%, with restored grip strength. By six months, on maintenance IVIG every four weeks, symptoms resolved fully, allowing return to work with accommodations. Clinician-assessed outcomes included full resolution of sensory and motor deficits. No important follow-up diagnostic tests were needed beyond routine monitoring. Intervention adherence was excellent, with good tolerability (no adverse events reported). No unanticipated events occurred, and no UC flare was noted during therapy.

## Discussion

This case illustrates a rare and atypical presentation of CIDP as an extraintestinal manifestation of UC, characterized by isolated distal fingertip paresthesia and weakness in the upper limbs without lower extremity involvement. While PN in IBD is documented, its incidence remains low, with studies reporting a prevalence of 1.9%-3.6%, often underdiagnosed due to subtle or overlapping symptoms [5]. In UC specifically, PN tends to be sensory or sensorimotor, axonal more than demyelinating, and frequently associated with disease activity or treatments [10]. However, demyelinating forms like CIDP are exceptional, with recent reports highlighting shared autoimmune pathways, possibly involving T-cell dysregulation or anti-neural antibodies triggered by gut inflammation [4].

The patient's occupational history as a factory worker initially suggested repetitive strain or entrapment neuropathy, such as CTS, which was ruled out. The bilateral, symmetric fingertip-restricted symptoms deviated from classic CTS (median nerve distribution) or diabetic neuropathy (stocking-glove pattern starting in the feet). Despite diabetes, the absence of lower limb symptoms, controlled glycemia (HbA1c 6.8%), demyelinating electrodiagnostic pattern (versus typical axonal in diabetic neuropathy), and rapid response to IVIG (unlike the poor immunosuppressive response in diabetic cases) argued against a primary diabetic etiology, favoring UC-related autoimmunity. Elevated inflammatory markers (ESR, CRP) and positive IgM anti-GM1 antibodies supported this reasoning. While anti-GM1 antibodies are more classically linked to multifocal motor neuropathy or Guillain-Barré syndrome variants, they can occur in CIDP, particularly in cases with motor involvement, and here served as supportive evidence of an immune-mediated process rather than a defining feature or incidental finding. The antibody positivity influenced the decision to pursue immunomodulatory therapy promptly, given the autoimmune context of UC. Diagnostic delay here - over a year - reflects systemic issues: occupational health focused on work-related causes, while public care overlooked neurologic workup. Literature echoes this, with PN often presenting insidiously in IBD, sometimes as the inaugural sign in pediatric cases [11]. Electrodiagnostic studies were pivotal, showing demyelination confined to upper limbs, an unusual topographic restriction that makes this case report-worthy. Most IBD-PN involves multifocal or generalized patterns, and isolated upper limb CIDP is rare, potentially due to selective vulnerability of distal nerves in autoimmune attacks. In terms of pathophysiology, intestinal immune dysregulation in UC may trigger peripheral myelin autoimmunity through mechanisms such as molecular mimicry, where gut microbiota dysbiosis leads to cross-reactivity between microbial antigens and neural gangliosides or myelin proteins, resulting in T-cell-mediated demyelination [12].

Therapeutically, IVIG prompted rapid improvement, consistent with CIDP's responsiveness to immunomodulation, unlike axonal forms that may require longer recovery [2]. This contrasts with diabetic neuropathy, which responds poorly to immunosuppression. Strengths of our approach include comprehensive exclusion of alternative causes and multidisciplinary input; limitations include lack of cerebrospinal fluid (CSF) analysis (patient refusal), which could have shown albuminocytologic dissociation, and absence of sural nerve biopsy, though not essential for diagnosis. This report emphasizes multidisciplinary collaboration - occupational, gastroenterology, and neurology - to unravel multifactorial neuropathies. In factory workers with IBD, clinicians should consider extraintestinal autoimmunity early, especially with atypical presentations, to avert functional decline.

The primary takeaway lessons are that CIDP can present atypically in UC with restricted upper-limb symptoms, and early electrodiagnostic and serologic evaluation (e.g., anti-GM1) facilitates timely immunomodulatory therapy, preventing disability. This case demonstrates an uncommon variant of CIDP associated with UC, presenting with highly restricted sensory and motor symptoms in the distal fingertips of the upper limbs, which initially evaded diagnosis due to overlapping comorbidities and occupational factors. The successful and rapid response to intravenous immunoglobulin therapy underscores the critical role of autoimmunity in driving the neuropathy, rather than the patient's concurrent well-controlled diabetes, and highlights the importance of immunomodulatory interventions in managing such conditions. Clinicians treating patients with inflammatory bowel disease should maintain a high index of suspicion for neurologic complications, even when presentations are atypical and limited to specific anatomic regions, as early electrodiagnostic testing and serologic evaluation for markers like anti-GM1 antibodies can facilitate timely diagnosis and prevent long-term disability. By advocating for comprehensive neurologic assessments in IBD patients exhibiting unusual paresthesia or weakness, this report contributes to greater awareness of these underrecognized extraintestinal manifestations, ultimately improving patient outcomes through proactive multidisciplinary care and avoiding diagnostic delays that could lead to irreversible nerve damage.

Patient perspective

The patient expressed relief at the resolution of his symptoms, noting that the treatment greatly improved his ability to perform daily tasks and work duties. He appreciated the thorough evaluation that addressed his concerns after initial delays.

Informed consent

Informed consent was obtained from the patient for publication of this case report.

## Conclusions

This case highlights an atypical presentation of CIDP in a patient with UC, characterized by sensory and motor symptoms restricted to the distal fingertips. Despite concurrent diabetes, the upper-limb-restricted distribution, demyelinating electrodiagnostic pattern, and rapid response to intravenous immunoglobulin confirmed an autoimmune etiology linked to UC, rather than diabetic neuropathy. Clinicians should suspect autoimmune neuropathies in IBD patients with unusual neurologic symptoms, as early electrodiagnostic testing and serologic evaluation for markers like anti-GM1 antibodies can prevent diagnostic delays, guide effective immunomodulatory therapy, and avert long-term disability.
